# Uncovering Spatiotemporal Heterogeneity of High-Grade Gliomas: From Disease Biology to Therapeutic Implications

**DOI:** 10.3389/fonc.2021.703764

**Published:** 2021-08-05

**Authors:** Andrea Comba, Syed M. Faisal, Maria Luisa Varela, Todd Hollon, Wajd N. Al-Holou, Yoshie Umemura, Felipe J. Nunez, Sebastien Motsch, Maria G. Castro, Pedro R. Lowenstein

**Affiliations:** ^1^Department of Neurosurgery, University of Michigan Medical School, Ann Arbor, MI, United States; ^2^Department of Cell and Developmental Biology, University of Michigan Medical School, Ann Arbor, MI, United States; ^3^Rogel Cancer Center, University of Michigan Medical School, Ann Arbor, MI, United States; ^4^Laboratory of Molecular and Cellular Therapy, Fundación Instituto Leloir, Buenos Aires, Argentina; ^5^School of Mathematical and Statistical Sciences, Arizona State University, Tempe, AZ, United States

**Keywords:** glioblastoma multiforme, heterogeneity, tumor microenvironment, dynamic, spatial resolution, deep learning, precision oncology

## Abstract

Glioblastomas (GBM) are the most common and aggressive tumors of the central nervous system. Rapid tumor growth and diffuse infiltration into healthy brain tissue, along with high intratumoral heterogeneity, challenge therapeutic efficacy and prognosis. A better understanding of spatiotemporal tumor heterogeneity at the histological, cellular, molecular, and dynamic levels would accelerate the development of novel treatments for this devastating brain cancer. Histologically, GBM is characterized by nuclear atypia, cellular pleomorphism, necrosis, microvascular proliferation, and pseudopalisades. At the cellular level, the glioma microenvironment comprises a heterogeneous landscape of cell populations, including tumor cells, non-transformed/reactive glial and neural cells, immune cells, mesenchymal cells, and stem cells, which support tumor growth and invasion through complex network crosstalk. Genomic and transcriptomic analyses of gliomas have revealed significant inter and intratumoral heterogeneity and insights into their molecular pathogenesis. Moreover, recent evidence suggests that diverse dynamics of collective motion patterns exist in glioma tumors, which correlate with histological features. We hypothesize that glioma heterogeneity is not stochastic, but rather arises from organized and dynamic attributes, which favor glioma malignancy and influences treatment regimens. This review highlights the importance of an integrative approach of glioma histopathological features, single-cell and spatially resolved transcriptomic and cellular dynamics to understand tumor heterogeneity and maximize therapeutic effects.

## Introduction

Glioblastoma (GBM) is the most common malignant primary brain tumor in adults, occurring most commonly in the 6^th^ to 7^th^ decade of life (1). GBM is classified by World Health Organization (WHO) as an astrocytic grade IV tumor, which commonly presents as a heterogeneously enhanced mass by neuroimaging. Microvascular proliferation, hypercellularity, nuclear atypia, pseudopalisades, cellular pleomorphism, and necrosis are hallmarks of GBM histopathology (2).

The prognosis for GBM is relatively poor and universally fatal, with a median overall survival of approximately 16 months from the time of diagnosis. O^6^-methylguanine-DNA methyl transferase (*MGMT*) promotor methylation is detected in about a third of GBM, and is prognostic of a better survival outcome, and predictive of better treatment response to alkylating chemotherapy. Isocitrate dehydrogenase 1 (IDH1) mutations, which are associated with more favorable outcome, represent a new tumor group termed ‘adult-type, diffuse glioma, IDH-mutant, astrocytoma, grades 2-4’, while glioblastoma is now reserved to the ‘adult-type, diffuse glioma, IDH1 wildtype’ (3–6). The current standard of care for GBM utilizes methods that are agnostic of molecular GBM phenotypes. They comprise an initial, maximally safe surgical resection, followed by conformal radiotherapy with concurrent oral temozolomide chemotherapy, followed by adjuvant temozolomide therapy. In addition, the use of tumor treatment fields has been introduced to the treatment of adult diffuse gliomas, though it is not considered part of standard of care (7, 8). Historically, each of the standard of care measures only adds a few months to survival. Although bevacizumab improves progression free survival, there is no evidence, at this time, that standard of care second line treatment improves overall survival (9, 10).

Advancement in GBM treatments is urgently needed; however, treating GBM faces numerous challenges due to, but not limited to, temporal and spatial tumor heterogeneity, altered cellular metabolism, and the unique immunosuppressive glioma microenvironment (11, 12). Immunotherapies and molecularly targeted personalized medicine have recently advanced the field of oncology in many cancer types; however, targeted agents against recurrent EGFR mutations and immune checkpoint inhibitors have so far not improved overall survival for GBM patients (13–19).

Moreover, assessing the treatment response holds significant importance to developing better GBM treatments. However, it can be quite difficult to differentiate tumor progression from inflammatory or necrotic changes associated with treatment, such as chemoradiation and immunotherapy, making neuroradiographic assessment suboptimal in these cases (20, 21). The blood-brain-barrier hinders drugs from reaching the tumor site, and also limits the utility of liquid biopsy (22). The lack of optimal surrogate markers of survival to effectively assess treatment efficacy is a paramount challenge the neuro-oncology community faces when evaluating potential new therapies (23). These challenges suggest that advanced, integrated histological, cellular, and molecular characterization with spatial resolution can provide insights for therapeutic interventions and predict clinical outcomes for GBM patients. Herein, we will review the recent advances made towards these integrated approaches.

## Molecular Genetics and Epigenetic Alterations in Glioma

GBMs differ in histologic features, malignancy grade, and molecular alterations. Recently, the presence and distribution of genetic/epigenetic alterations have been added as criteria to classify gliomas, refining the histological WHO classification, which previously defined these tumors as glial in origin (24–26). Recurrent IDH1 point mutations, which have been identified as contributors to gliomagenesis (27, 28), is used to classify gliomas and represents a major division of mutant IDH1 gliomas from wild-type-IDH1 (wt-IDH1) gliomas. wt-IDH1 gliomas, WHO grade IV, high grade gliomas (HGG) (12, 24, 29), present with several genomic alterations and higher somatic mutation frequency versus low grade gliomas (LGG) (30, 31). In adults, wt-IDH1 gliomas retain ATRX activity, and typically co-exhibit *TP53* and *TERT* promoter (*TERT*p) mutations. In addition, wt-IDH1 gliomas can harbor alterations in regulators of the RTK-RAS-PI3K signaling cascade, including EGFR amplification, as well as mutations or deletions to tumor-suppressor genes *PTEN* and *CDKN2A/B*, and alterations to chromosomes 7 and 10 (12, 24, 25, 31). IDH1 mutation, usually at arginine 132 (R132H), occurs in the vast majority of diffuse LGG (WHO grade II), and occurs also in a LGG that has recurred as GBM (WHO grade IV) (29, 32–35). IDH1-R132H, which is associated with better prognosis in glioma, catalyzes 2-hydroxyglutarate production, prompting epigenetic reprogramming of the glioma transcriptome (29, 32, 36–39).

A subgroup of adult-type diffuse mutant IDH1 gliomas which harbor 1p/19q chromosomal co-deletions (1p/19q-codel) and *TERT* promoter mutation are now classified as oligodendrogliomas (6, 40). Epigenetics alterations are a remarkable feature of gliomas with clinical significance. DNA methylation in CpG islands define the CpG island methylator phenotype (G-CIMP), a hallmark of mutant-IDH1 glioma, which is linked to better prognosis (41, 42). On the other hand, demethylation in *CXCR4*, *TBX18*, *SP5*, and *TMEM22* genes are related with tumor initiation and progression in GBM (43). Analyzing methylation profiles of TCGA data identified DNA methylation clusters designated subtypes LGm1 to LGm6, which were linked to molecular glioma subclasses and WHO grades (32). Also, methylation of CpG islands in the MGMT promoter predicts a better response to DNA alkylating agents (44). Recently, a novel methylation subgroup of IDH-WT GBM was introduced. This group differs from known molecular subgroups in terms of methylation and copy number profile with a distinct histological appearance and molecular signature (45).

In addition, different histone mutations are associated with pediatric brain tumors. Various studies have shown a high frequency of two-point mutations in the genes of the histone variants H3.3 “H3F3A”, and to a lesser extent H3.1 “HIST1H3B”, which result in substitution of lysine at position 27 with methionine (K27M) or glycine at position 34 with arginine or valine (G34V/R). Further reports highlighted the association of K27M mutation with midline gliomas (MLG) and G34V/R mutation with gliomas of the cerebral hemispheres (46–48). In this context, epigenetic modifications to histone tails by methylation or acetylation in gliomas impact gene expression and, therefore, tumor characteristics (38, 49, 50). Identification of these alterations have been useful for predicting prognosis of glioma patients (51) and for developing therapeutics agents targeting regulators of histone modifications, such as DNA methyltransferase (DNMT) inhibitors and histone deacetylase inhibitors (HDACIs) (52).

As a consequence of the genetic alterations that classify gliomas, significant signaling pathways are altered. This includes activation of the growth factor receptor tyrosine kinase (RTK) pathways as result of PDGF and EGFR overexpression (53, 54). The frequent activation of RAS, PI3K/PTEN/AKT, RB/CDKN2A-p16INK4a, and TP53/MDM2/MDM4/CDKN2A-p14ARF pathways are implicated in glioma proliferation (55, 56). On the other hand, the anaplastic features of HGG/GBM can be boosted by NOTCH signaling activation, which is related with hypoxia and PI3K/AKT/mTOR and ERK/MAPK pathways (57). Other alterations in glioma cell signaling include metabolic (58), cell differentiation (59), and DNA repair (38, 60) pathways, all with the therapeutic implications.

## HGG Intertumoral and Intratumoral Molecular Heterogeneity

**Figure 1:**
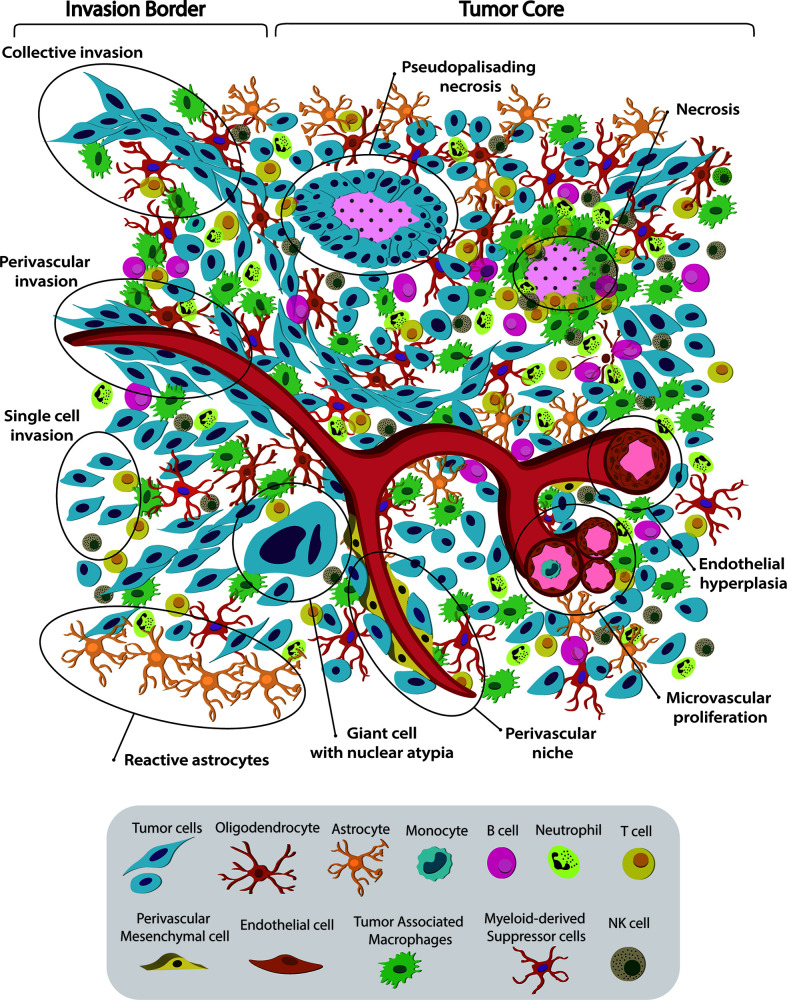
Spatiotemporal complex intratumoral heterogeneity of GBM. GBM intratumoral heterogeneity at the histological, cellular and dynamic level is illustrated. The schematic representation of the gliomas TME highlights the spatio-temporal heterogeneity at the histological, dynamic, and cellular level. We indicate various hallmarks of GBM. (1) Pseudopalisading necrosis in GBM is characterized by garland-like organization of tumor cells at the edge of areas of tumor necrosis. Glioma cells migrate away from hypoxic regions and invade into healthy tissue at the infiltrating edge. (2) Endothelial hyperplasia represents the vascular lesions characterized by the proliferation of endothelial cells. Glomeruloid vessels and extensive endothelial multilayering result from the endothelial hyperplasia characteristic of GBM. (3) Microvascular proliferation appears as glomeruloid tufts of multilayered endothelial cells together with smooth muscle cells and pericytes. VEGF release from the surrounding necrosis tissue acts on nearby vessels to cause vascular hyperplasia, including microvascular proliferation. (4) Scattered large pleomorphic glioma cells represent multinucleated giant cells with generalized nuclear atypia. (5) Poorly vascularized regions of the tumor become hypoxic and necrotic. At the dynamic level GMB displays different migratory patterns. (6) The tumor–brain interface is characterized by an invasive edge that harbors invasive glioma cells that migrate along white matter tracts or extracellular matrix fibers to infiltrate the brain either as collective invasion (i.e., connected elongated cells infiltrating the brain parenchyma), or (7) Single-cell invasion characterized by amoeboid movements, weak intercellular adhesions, and random movement. (8) Glioma cells are shown to also invade collectively using the perivascular space. Perivascular glioma cells quickly invade the perivascular space as a conduit for invasion. Bottom panel shows the striking cellular heterogeneity of GBM, being composed of both neoplastic cells and nonmalignant cells. It includes several phenotypes of tumor cells, such as rounded cells and mesenchymal-like cells, as well as nonmalignant cells, that form the tumor microenvironment (TME) and make up 50% of the tumor mass. TME is composed of normal brain residents: neurons, astrocytes, oligodendrocytes and microglia; endothelial cells from the vasculature, surrounded by perivascular-mesenchymal cells; and immune system infiltrating cells. 95% of the TME are tumor associated macrophages (TAM), derived either from circulating monocytes or microglia. The remaining 5% are mainly dendritic cells, with smaller contributions of T cells, B cells, NK cells and neutrophils. Understanding tumor heterogeneity composition allows to employ better antitumor therapies.

HGG/GBM are characterized by high intertumoral and intratumoral heterogeneity. This heterogeneity is observed at different inter-related levels (histological, cellular and molecular) and is one of the main features that hinders tumor treatment (**Figure 1**). Molecular unsupervised transcriptome analysis of GBM revealed different tumor clusters, highlighting the prominent intertumoral heterogeneity. Different studies over the past 15 years have attempted to classify GBM into molecular subtypes. Back in 2006, Phillips et al. reported the molecular gene expression profile of 76 HGGs, defining signatures from a set of 35 genes, which characterized 3 different subtypes: Proneural, Proliferative, and Mesenchymal. They found a correlation between molecular subtypes and histological tumor grade. Also, Mesenchymal and Proliferative tumors showed a markedly inferior prognosis compared to Proneural (61). Subsequent studies carried out by Verhaak et al. used integrated, multidimensional genomic data and DNA copy number to define a more robust gene expression-based molecular GBM classification into 3 confirmed subtypes with a signature from 210 genes per subtype (53). Overall, aberrations and gene expression of *EGFR*, *NF1*, and *PDGFRA* define Classical, Mesenchymal, and Proneural subtypes, respectively. Specifically, the Classical subtype exhibited chromosome 7 amplification associated with high-level EGFR amplification. This subtype also lacked distinct additional genetic abnormalities in TP53, NF1, PDGFRA, or IDH1, but affected expression of genes, such as FGFR3, PDGFA, EGFR, AKT2, and NES. The Mesenchymal subtype displayed focal hemizygous deletions of a region at 17q11.2, containing the gene NF1. This subtype was associated with greater necrosis and inflammatory infiltrates, which was linked to higher expression of tumor necrosis factor and NF-kB pathway genes, such as TRADD, RELB, and TNFRSF1A. Some of the most relevant differentially expressed genes of Mesenchymal tumors were CASP1/4/5/8, ILR4, CHI3L1, TRADD, TLR2/4, and RELB, among others. The Proneural subtype was defined by PDGFRA and TP53 alterations and IDH1point mutations and differential expression of DLL3, NKX2-2, SOX2, ERBB3, and OLIG2 (53).

Using DNA methylation profiles from 396 GBMs, Brennan et al. in 2013 identified six methylation clusters. They found that Cluster M1 (60%) was enriched in Mesenchymal subtype, Cluster M3 (58%) was enriched in Classical subtype, and the G-CIMP cluster was enriched in Proneural subtype. They observed that the Mesenchymal subtype expressed higher levels of endothelial markers, such as CD31 and VEGFR2, in concordance with Phillips et al. (61) and inflammation markers, such as fibronectin and COX2. On the other hand, the Proneural subtype was associated with somatic mutations to genes such as IDH1, TP53, ATRX, and MYC, and the Classical subtype with EGFR amplifications or mutations (31).

Lastly, in 2017, Wang et al. postulated GBM-specific intertumoral heterogeneity, and defined 3 tumor-intrinsic transcriptional subtypes from transcriptomic analysis of wt-IDH GBM samples, derivative neurospheres, and single-glioma-cell gene expression profiles (62, 63). Subtypes were designated as Proneural, Mesenchymal, and Classical using a 50-gene expression signature per subtype, which represented a 42 to 54% overlap with previous studies (53). The 50-gene expression signature by subtype could be summarized by the most relevant genes from each group. The Mesenchymal subtype overexpresses BCL3, TGFBI, ITGB1, LOX, COL1A2, VDR, IL6, and MMP7, the Proneural subtype has increased expression of GARBR3, HOXD3, ERBB3, SOX10, CDKN1C, PDGFRA, HDAC2, and EPHB1. Finally, the Classical subtype was characterized by overexpression of PTPRA, ELOVL2, SOX9, PAX6, CDH4, SEPT11, MEOX2, and FGFR3, among others.

A new study by Garofano, L. et al. postulates a novel pathway-based stratification of GBM that uncovers new subtypes with potential prognostic relevance, namely mitochondrial (MTC), glycolytic/plurimetabolic (GPM), proliferative/progenitor (PPR), and neuronal (NEU) (64).

In another study, Neftel et al. showed that glioma subtypes are associate with a set of cellular states that define 4 different groups: NPC-like (neural progenitor like), OPC-like (oligodendrocyte progenitor like), AC-like (Astrocyte like) and MES-like (mesenchymal like). The frequency of each steady-state is modulated by specific genetic modifications (CDK4, PDGFRA, EGFR and NF1); in addition, each single tumor can contain a diversity of states maintained by cellular plasticity (65).

Although similarities and discrepancies surround glioma subtype classification, the Mesenchymal subtype is one of the steadiest subtypes, when analyzing human GBM tissues, GBM xenograft models, and derivative GBM stem cells (53, 61, 66, 67).

In addition to the vast molecular intertumoral heterogeneity, GBM also exhibit high heterogeneity within the same tumor mass, showing histologically and molecularly dissimilar areas (**Figure 1**). Research studies using tumor sampling from different anatomical locations demonstrated that 60% (6/10) of tumors presented two or three different subtypes within the same tumor (68). Other studies showed that molecular subtypes correlate with histological features. Mesenchymal tumor was associated with hypoxic and perinecrotic areas and high microvascular proliferative zones, while Classical was related to vascular and invasive zones. Tumors with these two characteristics had the worst prognosis (69).

Single-cell RNA-Seq (scRNA-Seq) analysis has emerged as an important approach to dissect the cellular and molecular profile of complex tumors compared to bulk conventional analysis. scRNA-Seq has yielded insights into phenotypic and genotypic differences resulting from tumor cells, the relation with the neural lineages, and the tumor microenvironment, and subpopulations of transformed cells in these extremely heterogeneous tumors (63, 70–72). Analysis of scRNA-Seq suggested that GBM consist of a combination of tumor cells with different GBM subtype footprints (63). Patel et al. analyzed intratumoral heterogeneity by single-cell full-length transcriptomes (SMART-Seq) of isolated cells from five freshly resected human wt-IDH/EGFR amplified GBM depleted of CD45+ cells. They observed a genetic correlation between individual cells and transcriptional intratumoral heterogeneity within the same tumor. They also observed mosaic protein expression of common signaling pathways affected in GBM, such as EGFR, PDGFRA, PDGFA, FGFR1, FGF1, NOTCH2, and JAG1. Interestingly, all tumors contained heterogeneous combinations of individual cells corresponding to different TCGA defined subtypes. They observed that intratumoral subtype heterogeneity imparted significant insights into GBM biology and prognosis, where extensive heterogeneity was associated with reduced survival. Tumors highly heterogeneous for different subtypes or displaying Mesenchymal signatures had poorer outcome than pure Proneural GBM (63). On the other hand, Wang et al. reported multiple activation of different subtypes associated with intratumoral heterogeneity. They suggested that only 8% of the TCGA samples activated more than one transcriptional subtype, displaying low simplicity scores, while GBM samples with a single subtype had higher simplicity scores. Using this approach, they demonstrated that samples with high simplicity scores had significant survival differences between Mesenchymal and non-Mesenchymal tumors. They concluded that the intratumoral heterogeneity at single-cell level is captured in the transcriptional signature of the bulk tumor (66).

A recent study suggested that tumoral classification pays little attention to the importance of existing intratumoral heterogeneity. They focused on regional architecture of the tumor by analyzing different tumor areas using 9 immunoreactivity biomarkers relevant for GBM. They found that 3 of the 5 pathophysiologically relevant clusters, transformed neuronal, highly proliferative, and mesenchymal stem cell regions, correlated with the 3 tumor subtypes described by Phillips et al. Particularly the Mesenchymal subtype was characterized by high vimentin and nestin expression levels (73). All together, these studies highlight the complexity of GBM molecular signatures and emphasize the importance of considering intratumoral heterogeneity to understand tumor growth and invasion, and develop novel antitumor strategies.

## Glioma Tumor Microenvironment and Cellular Heterogeneity

Gliomas are a complex composition of both malignant and nonmalignant cells. Nonmalignant cells, including microglia, astrocytes, macrophages, lymphocytes, endothelial, and other cells, collectively constitute the tumor microenvironment (TME), making up ~50% of GBM tumor mass as shown in **Figure 1** (71). The vast majority of GBM infiltrate can be classified as either macrophage or microglia (~95%), with the remaining population comprised primarily of dendritic cells (~4.5%) (71). Darmanis et al. found that transcriptionally distinct immune cells residing in the core increased tumor growth, survival, and invasion by inhibiting inflammation, increasing angiogenesis, and extracellular matrix remodeling (71). Microglia are the predominant resident immune cells in the healthy brain; however, under pathological conditions, brain parenchyma recruits circulating monocytes, which differentiate into macrophages (74, 75). Tumor-associated macrophages (TAM) play key roles in promoting invasion, angiogenesis, metastasis, and immune suppression (76). They can originate from two distinct lineages: tissue-resident microglia (CD45^lo^ MG-TAM) or monocytes recruited from peripheral circulation (CD45^hi^ M-TAM) (77–79). LGG tend to have more MG-TAM, while HGG are enriched in M-TAM (80). Recent work has described that in GBM TAMs within the tumor core mostly originate from the bone marrow derived pool whereas those in the tumor periphery are largely derived from microglial cells (81). These findings correlate with transcriptomic data (71) and reviewed in (79). TAM populations can also be subdivided into activation state phenotypes: Unstimulated M0, classically activated M1, and alternatively activated M2 (17, 74). The M1 phenotype is anti-tumorigenic and is present at lower levels in GBM infiltrate, while the M2 phenotype is pro-tumorigenic and more abundant, correlating with shorter survival (82). It has been shown that resident microglia are crucial modulatory cell population playing a central role in regulation of vascular homeostasis and angiogenesis and represent an alternative source of pro-angiogenic growth factors and cytokines (79, 83). CXCL2 is expressed in several cell types present in GBM such us endothelial cells, glioma cells, T cells, mast cells and myeloid cells, and its expression level has been correlated with GBM aggressiveness (84). Isolated microglia/macrophages from glioma produce a variety of pro-angiogenic molecules as well as high level of CXCL2 (83). CXCL2/IL8/CXCR2 axis has showed to be involved in maintaining GBM angiogenesis (85, 86). The CXCR2 antagonist SB225002 has shown inhibition in tumor growth, and led to reduced number of TAMs as well as tumor vessels (85, 86). Malignant cells recruit microglia and macrophages to the tumor, where they acquire an M2 phenotype and contribute to an immunosuppressive TME. One of the main factors recruiting TAM is the chemo-attractant, colony-stimulating factor (CSF), which is also a critical for macrophage function. Attenuating the interaction between CSF-1 and its receptor by employing target inhibitors reduces TAM numbers at the tumor site and impairs glioma invasion (87).

Although they have an abundance of TAM, gliomas are defined as immunogenically “cold” because they have low levels of infiltrating T cells (17, 88). Lymphocyte infiltrate present in the TME are CD4+ T helper, CD8+ T cytotoxic, and Tregs, with CD4+ cells more numerous than CD8+ (89). High Treg levels in GBM suppress the function of antigen-presenting cells and inhibit T cell proliferation, contributing to tumor evasion (90). Tregs may be immunosuppressive by employing immune checkpoints molecules, such as CTLA-4 and PD-L1 (17, 19, 91, 92). Also, recent work showed that inhibition of the CLEC2D-CD161 pathway may provide synergistic therapeutic benefit when combined with PD-1 blockade by enhancing the anti-tumor function infiltrating T cells in GBM of distinct T cell populations (93).

Myeloid-derived suppressor cells (MDSC) are found extensively in GBM TME (94). They are a heterogeneous population of immature myeloid cells formed from myeloid progenitors and macrophage, granulocyte, and dendritic cell precursors. However, MDSC do have some common features, such as their myeloid origin, immature state, and, most importantly, the ability to convert immune responses from a Th1 to a Th2 phenotype, which potently inhibits CD4+ and CD8+ T cells and fosters an immunosuppressive TME (95). Inhibiting COX2 reduces MDSC recruitment and increases cytotoxic T cell levels (96).

Elevated tumor-associated neutrophil (TAN) infiltration correlates with lower survival, suggesting that neutrophil infiltrate contributes to immunosuppression (97), and subsequent tumorigenesis and tumor growth. Elevated neutrophil CXCL8 expression boosts recruitment, and is found in high levels in gliomas (98). Disrupting the interaction of CXCL8 with its receptors, CXCR1 and CXCR2, is a possible approach for dismantling neutrophil infiltration and its associated immune suppression. In addition to TAM, MDSC, TAN, and Tregs, Bregs also suppress the immune response in GBM by interacting with other TME cells to augment immunosuppression (99). Glioma cells can induce a phenotype switch from B cells to Bregs, which contributes to Tregs recruitment and suppression of CD8+ T cells (100, 101).

There are also non-immune cell components of the GBM TME, which contribute to tumor progression. A common histologic feature of glioma is reactive astrocytosis, in which tumor-associated astrocytes are more proliferative, have JAK–STAT pathway activation, and CD274 expression (102). Astrocytes, as wells as microglia, secrete anti-inflammatory cytokines, contributing to an immunosuppressive environment (102). A pro-tumorigenic function has also been described for neurons, by either paracrine or autocrine mechanisms (103), as well as through functional synaptic integrations (104). Even though oligodendrocytes are detected in relatively high numbers by scRNA-Seq of glioma clinical samples, their role in glioma pathology has yet to be determined.

Stromal components, such as endothelial cells, and pericyte/mesenchymal stem cells (MSC), also play a role in tumor formation and progression. MSC are a small population characterized by self-renewal, expression of stemness markers, and multi-lineage differentiation properties (105). Tumor cells hijack neural development mechanisms, shifting MSC into glioblastoma stem cells (GSC), which possess tumor-propagating potential and are resistant to radiotherapy and chemotherapies (105, 106). Since MSC cells share expression markers with pericytes and are mainly localized around blood vessels (107), it is difficult to differentiate MSCs from pericytes (108). Up till now, there is no exclusive set of expression markers that differentiates MSC from pericytes, making it difficult to distinguish between them.

As outlined in this section, the interactions between glioma cells and constituents of the TME play key roles in tumor growth and progression. A deeper understanding of the dynamics of these interactions would bring us a step closer to designing effective treatments.

## Tumoral Dynamic Heterogeneity Patterns Across Histologic Features

Gliomas are characterized by intratumoral heterogeneity and diffuse invasion into the healthy parenchyma. In doing so, gliomas use various motility patterns, *i.e*., single cell invasion or collective invasion (**Figure 1**) (109–113). Tumor growth and invasion is usually considered to be a stochastic. However, whether tumor growth actually results from random processes, or whether gliomas self-organize to promote tumor growth and invasion is not well understood. Thus, the existence of organized dynamic structures in tumors, and what role they may play in tumor progression remains poorly elucidated (114). We recently characterized the complex dynamics of glioma cells in both the tumor core and at the tumor invasive borders, using mouse glioma explants from genetically engineered mouse models (111, 113). We recently found that collective motion of tumor cells can be identified histologically as fascicles of aligned spindle-like and mesenchymal-like tumor cells. For simplicity, we propose to refer to these fascicles as oncostreams. Together with their capacity for collective motion, our data indicate that they likely contribute to tumor malignant behavior. Thus, we interpret oncostreams to be histological structures that represent areas of collective motion (113). As our data indicate that oncostream density correlates with tumor malignancy, we suggest that they are characteristic pathological components of gliomas. Oncostreams display two main types of collective motion, as defined elsewhere by us (112): (i) streams (↑↓) = cells move in both directions, (ii) flocks (↑↑) = cells move mostly in one direction. Cells that move without a preferred direction are defined as swarms and are histologically identified as areas of round cells. We recently showed, using agent-based mathematical modeling, that interactions between individual cells are sufficient to produce these large-scale patterns of collective motion (112). Collective motion patterns have been observed during normal development and also in pathological conditions, such as epithelial to mesenchymal transitions in cancers, followed by metastasis to distant organs (115–117). Directionally correlated cell movement within the tumor core have been also observed in recent studies using *ex vivo* explants of spontaneous intestinal carcinoma. Staneva et al. provided detailed mathematical support for the existence of dynamic patterns, such as *currents* and *vortices*. Their currents are homologues to our *flocks*, since cells move in one single direction in both descriptions (118). Equally, studies of *in vivo* motility of human glioma cell invasion within immune-suppressed animals, indicate complex motility patterns at the tumor border (118). Interestingly, these authors determined that cells can actually move towards and away from the tumor, using two types of invasion patterns at the glioma border, the *invasive margin* of multicellular invading groups of cells, and the *diffuse infiltration* of single cells. *Swarms*, in our descriptions, correspond to diffuse infiltration, since these cells present with an increased speed and less directionality in both studies, whereas the invasive margin corresponds to our directional collective motion patterns (119). The role of collective dynamic patterns within glioma tumors has not been addressed in detail so far. A better understanding of glioma dynamic heterogeneity, taking into account its constituent histological features and their underlying molecular basis, are essential to provide a more accurate picture of gliomas. We believe that the eventual pharmacological disruption of collective glioma dynamic patterns will inhibit glioma growth and progression, and will become a novel treatment approach.

## The GBM Molecular Landscape: Current and Futures Perspectives in Methodologies to Analyze GBM With Spatial Resolution

**Figure 2:**
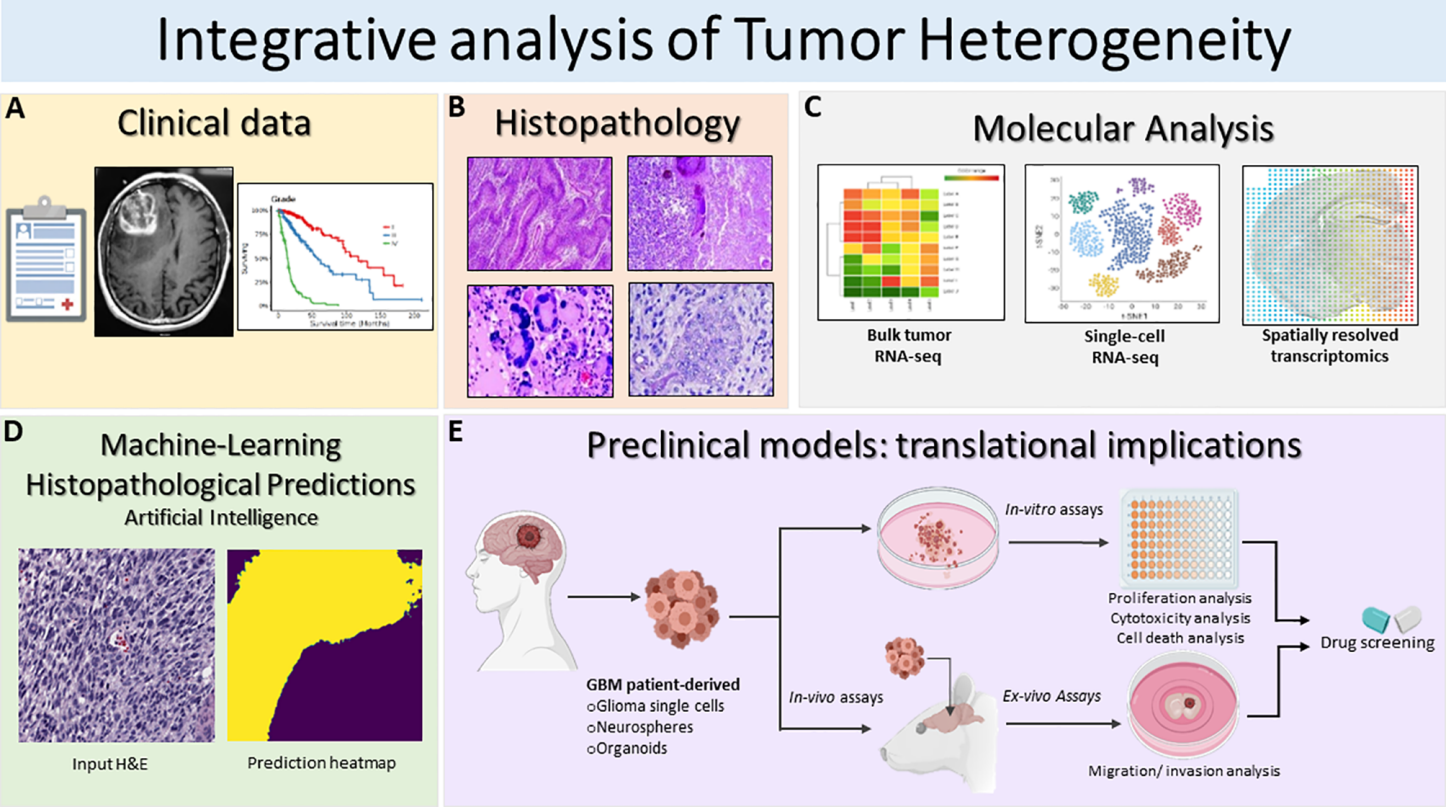
Schematic illustration of the integrative translational preclinical and clinical workflow for translational implications. **(A)** First, clinical information is collected including the MRI and specific glioma grade type. **(B)** Following surgery, neuropathological studies are used for both histopathological assessment and investigation of intratumoral heterogeneity. The histopathology images in **Figure 2B** are modified from Figures 1.34A, 1.35A,B, 1.41A in Chapter 1 Astrocyte Tumors, in IARC, and Otmar D. Wiestler. WHO Classification of Tumours of the Central Nervous System: WHO Classification of Tumours of the Central Nervous System (4th Edition), edited by David N. Louis, World Health Organization, 2006 (120). **(C)** Next, multiregional studies of the surgical samples can be harnessed to determine molecular targets within GBM employing bulk RNA-seq, scRNA-seq and spatially resolved transcriptomics. scRNA-seq method examines heterogeneity at very detailed resolution. The precision treatments are assessed according to tumor heterogeneity evaluation and potential pharmacological sensitivities. **(D)** Prediction of histopathological features based on a novel artificial intelligence analysis of histological data will further aid clinical decisions. Furthermore, intratumoral heterogeneity assessment based on advanced imaging and machine-learning predictions should be carried out to monitor spatio-temporal heterogeneity dynamically and treatment. **(E)** GBM patient derived glioma cells, neurospheres and organoids can be exploited for preclinical modeling and perform pharmacological drug screening using *in-vitro* and *ex-vivo* assays. Patient derived organoids model the parental tissue and can be used to complement standard molecular pathology to understand mechanisms of resistance and can be applied to numerous functional assays such as: tumor cell survival, proliferation and self-renewal, and *ex-vivo* invasion/migration assays to identify pharmacological agent to target glioma invasion. Integrative GBM analysis attempts to improve predictive outcomes and treatments for GBM.

Molecular studies of bulk tumors or scRNA-Seq studies disclose the complex cellular and molecular heterogeneity of GBM, but lack the spatial dimension of tumor tissue. The spatial heterogeneity of glioma tumors is not just regulated by the mixture of genotypic profile of individual cells, but rather is shaped by the crosstalk between tumor and TME cells in different tumor areas. Understanding how the molecular heterogeneity relates to the classical histological GBM hallmarks would provide invaluable information for integrated characterization, diagnosis, and treatment (**Figure 2**).

In recent years, *in situ* spatially characterized tissue analysis using state of the art technology, such as spatial transcriptomics or multiplex protein expression, opened up new paths to study in greater detail the cellular and molecular heterogeneity in the context of intact tumor tissue including GBM (121, 122). These technologies span tissue laser capture microdissection (LCM) combined with *ex situ* RNA-Seq analysis, *in situ* DNA oligonucleotide barcoding followed by *ex situ* sequencing, and computationally assigned spatial information to expression and imaging methods based on fluorescence *in situ* hybridization (FISH) or *in situ* sequencing (ISS) (123). Methodology parameters, such as sample type and processing, number of detected genes, experimental throughput, and spatial resolution need to be considered before selecting the appropriate method.

Some studies using spatially resolved transcriptomic analysis demonstrated the importance of these technology for examining spatial heterogeneity of the glioma TME. Laser scanning microdissection and RNA-Seq analysis assigned genomic alterations and gene expression patterns to specific GBM histological hallmarks, including tumor infiltration, pseudopalisades cells around necrotic areas, and cellular tumor and microvascular proliferation (69). This Ivy Glioblastoma Atlas project (IvyGAP) combined spatial molecular information with histological features and the clinical database from the patients’ cohort, providing deeper understanding of tumor heterogeneity. Intratumoral microenvironment-specific expression from the IvyGAP atlas also advocated potential therapeutic avenues by identifying brain tumor initiating cells and target genes within individual anatomical regions (124, 125). Our recent study proposed an improved laser scanning microdissection methodology to study the gene expression pattern of multicellular mesenchymal-like structures within the glioma tumor core and at the invasion front (113, 125–128).

Novel studies have recently provided new perspectives in the analysis of proteomics, metabolomics, and lipidomics in different cancers (129–131). In GBM, Gularyan et al. describe developing the TOF-SIMS (time-of-flight secondary ion mass spectrometry) methodology to detect protein expression and metabolites in paraffin or frozen glioma sections with spatial resolution (132). This allowed morphological differentiation of diverse regions in patient-derived tumors, which correlated with clinically relevant data, *i.e*., tumor grade, survival, to study GBM. Employing these emerging methodologies that combine histopathology with next generation sequencing or metabolomics are essential for translational applications, which identify novel potential targets in glioma tumors. These approaches could generate a new understanding of glioma behavior, uncovering the heterogeneity in functions, dynamics, and interrelation of tumor cells with TME cells (**Figure 2**).

## Computational Deep Learning Analysis and Novel Imaging Technologies Applied to Tumor Heterogeneity

The recent breakthroughs in artificial intelligence (AI), specifically deep neural networks, have resulted in major advances in glioma radiomics and digital pathology. Given a sufficiently large amount of training data, deep neural networks can identify the optimal set of image features to achieve high performance on a specific task, such as image classification. For example, deep neural networks can classify tumors harboring isocitrate dehydrogenase-1/2 (IDH1/2) mutations versus wt-IDH1/2 from brain magnetic resonance imaging (MRI) (133, 134). Similar methods have been applied to diagnose 1p/19q co-deletion and *MGMT* promotor methylation status (135).

Over the previous decade, digital pathology has experienced a renaissance due to two major factors: (1) the availability of large, public pathology datasets (136) and (2) major breakthroughs in computer vision methods. The application of deep neural networks to the analysis and interpretation of whole-slide images (WSI) has ushered in a new era of digital pathology in cancer (137–140). Efficient whole-slide scanning and digital pathology tools have allowed for quantitative microscopic analysis of tumor heterogeneity (141). Tumor microscopy provides essential phenotypic and microenvironment features not characterized by molecular profiling or -omics data. The spatial relationships between tumor-associated stroma and tumor infiltration can be directly visualized at single-cell resolution using digital pathology (**Figure 2**).

Our group is currently investigating using optical microscopy and AI to rapidly characterize fresh glioma specimens (138, 142–144). By combining stimulated Raman histology, a rapid, label-free optical imaging method, with deep neural networks, we can automate glioma classification and grading in under 2 minutes. Moreover, we can detect regions of dense, viable tumor infiltration in primary and recurrent tumors.

## Glioma Heterogeneity and TME in Clinical Therapeutic Resistance

Within individual tumors, there is significant heterogeneity at the TME level, wherein unique spatial niches harbor numerous cell populations (65, 145–148). These niches are dynamic and adjust to environmental pressures, such as treatment. Indeed, recent data reinforces this adaptive remodeling within tumors. Neftel et al. showed that gene expression in GBM is driven by four different cellular states, which are dynamic and driven by genetic, epigenetic, and microenvironmental factors (65). Even unique genetic subclones were found to exist within all 4 cellular states. Longitudinal assessment of paired patient specimens has revealed unique patterns of clonal evolution with standard of care treatment, highlighting evidence that rare resistant subclones often exist within the initial tumors that are often responsible for treatment resistance (149, 150).

The clinical implication of such profound dynamic cellular and microenvironmental heterogeneity is vast. How does one target a tumor with various subtypes of dynamic gene expression wherein local TMEs maintain and protect tumor cells? With this understanding, it is not unexpected that single target therapies have largely failed in GBM. For example, EGFR alterations are common GBM driver mutations, most frequently as the EGFRvIII variant, which results in a detectable antigen. Clinical trial results of rindopepimut, a peptide vaccine targeting this variant, were disappointing and found that patients who progressed through treatment lost EGFRvIII expression (18, 151, 152). SRC and SRC family kinases (SFKs) have a broad and important role in numerous signaling pathways, which promote GBM tumor growth and invasion; however, a clinical trial assessing Dasatinib, a potent SFK inhibitor, failed to meet its clinical endpoint (153, 154). Similar results occurred from targeting KIT amplification or mutation with Imatinib (155, 156) and TGFβ inhibitors (157, 158), amongst others. The most well-known failure of a single targeted therapy is bevacizumab, a monoclonal antibody against the vascular endothelial growth factor (VEGF), which is highly expressed in GBM and associated with endothelial cell proliferation and angiogenesis (9, 10). Despite an initial imaging response, patient survival did not improve. On the other hand, there are targeted therapies that have shown some promising results, such as targeting BRAFv600e mutations, although using single agents often results in recurrence, which has led to targeting BRAF combined with MEK inhibition (131, 159). In spite of many new therapeutic approaches being in clinical trials, so far, unfortunately, none has shown efficacy in randomized control double blinded Phase III clinical trials (16, 17, 160)

Overall, results from targeted therapy have been disappointing, although there may be numerous reasons why certain therapies were unsuccessful. Despite positive findings in murine models, penetrance across the human blood brain barrier and activity within the brain at the clinical drug dosages are rarely validated. Perhaps the dosage required for penetration and efficacy in humans is not utilized, or not attainable. This further emphasizes the need for phase 0 studies assessing drug penetration and response in human studies. Furthermore, clinical trials may have failed to properly enrich patients likely to benefit; thus, identifying appropriate biomarkers may lead to better patient selection.

Nevertheless, the most likely failure of our current treatment strategies is a lack of understanding of the significant dynamic tumor heterogeneity, which drives therapeutic resistance. However, it is yet unclear how clinical therapeutics can be altered to target this intra and intertumoral heterogeneity. We must consider therapies that address multiple resistance pathways, including immune-based therapies that may target multiple tumor antigens (19, 161–163), or therapies targeting common metabolic and physiological pathways, which may improve chances of success (164, 165). Furthermore, greater effort in developing preclinical models and clinical studies to understand spatial heterogeneity, tumor recurrence, and evolutionary trajectories in GBM are vital (**Figure 2**).

## Precision Oncology for Gliomas: Targeting Spatial Heterogeneity

Cancer therapies have evolved from traditional chemotherapy and radiotherapy options to more personalized and focused approaches. We have seen remarkable progress in recent years, especially in pancreatic, prostate, and ovarian cancers. Precision oncology leverages genetic alterations and molecular markers present in the patient tumor to deliver a personalized therapeutic regimen (166). The progress of precision medicine essentially relies on identifying targetable biological features in tumors (167). This presents a significant challenge, especially for GBM, which is highly heterogeneous. Glioma cells vary in their morphology, underlying gene expression, and genetic mutational landscape (168). Consequently, any chosen therapeutic target may be expressed by most, but not all, cells, resulting in incomplete tumor eradication.

Mutant IDH status, *MGMT* promoter methylation status, BRAF mutation, and upregulated PI3K/AKT/mTOR signaling pathway have drawn attention as actionable alterations in LGG patients (35, 169–171). Adult brain tumors have seen some progress with precision medicine approaches, especially targeting BRAF, H3K27 demethylation, and NTRK fusions (170, 172, 173). Targeting DNA repair mechanisms with PARP inhibitors, and mutant IDH enzyme and gene fusions with appropriate inhibitors holds potential for treating GBM patients with such genetic alterations (171, 172, 174–176). Identification of several markers relevant to GBM diagnostics using liquid biopsies with NGS for circulating free DNA and/or circulating tumor cells could be used in molecular diagnosis of cytological specimens and potential administration of innovative precision therapy (177, 178).

Nevertheless, spatial and temporal heterogeneity is a critical challenge that the neuro-oncology field must address before precision oncology can be considered a viable option for brain tumor patients (62, 69, 179, 180). Spatial heterogeneity in GBM resected tumors is recognized in transcriptional atlases, where genomic alterations and gene expression patterns vary between the leading edge, infiltrating tumor, cellular tumor, pseudopalisading cells around necrosis, and microvascular proliferation regions (69). To explain the evolution of multiple GBMs (M-GBMs), Lee et al. proposed a multiverse model based on extensive bulk and single-cell RNAseq data (62). They demonstrated that M-GBMs are more genetically diverse than nearby tumors and genetic similarity between multiregional samples correlates with treatment response. Furthermore, enrichment of *PIK3CA* mutations in M-GBMs, as well as the effects of PAM inhibitors, which are more selective in patient-derived glioma cells. Their findings support the truncal target hypothesis, which states that truncal mutations can help guide more effective therapies (62). Recently, it has been shown within a single GBM tumor, that intratumoral spatial heterogeneity of Bruton’s Tyrosine kinase activity in tumor core versus edge cells showed distinct therapeutic responses (181).

Glioma-initiating cells (early-branched, ancestor-like tumor cells) at tumor edges receive signals from the tumor core, which promotes their malignancy (182–184). Evidence from several murine tumor models supports the Edge-to-Core progression theory (182). However, it is unclear if this hypothesis universally describes the development of primary GBM. Brain tumor cells at the edge reside in a distinct environment from the tumor core, interacting with various somatic cells, including neurons, astrocytes, vascular endothelial cells, and immune cells (104, 185–188). These tumor-associated somatic cells may contain cellular populations that can activate or suppress tumor cells. Multi-OMICs studies have established largely distinct signaling pathways activated in edge- and core-located tumor cells viz., Esm1/endocan, Bruton’s Tyrosine Kinase, nitrogen metabolism. Thus, developing spatially distinct therapeutic modalities for GBM is a critical challenge (181, 182, 189–191). Understanding the phenotypic complexities of patient tumor cells will necessitate molecular investigation to develop effective precision treatments for gliomas (**Figure 2**).

## Conclusions

Over the last few years, tumor heterogeneity has come to the forefront as a *bona fide* hallmark of tumor biology, including tumor dynamics, migration, and invasion. In the particular case of GBM, heterogeneity is present at the anatomical, histological, functional, molecular, vascular, and immune levels. The complex spatiotemporal structure of brain tumors is likely a major contributor to the difficulties of treating these tumors since treatments may not be equally effective across heterogeneous tumor areas. The presence of heterogeneity means that treatments should be tailored to target microenvironments, since the tumor cell characteristics and their microenvironments vary by tumor location. However, it has been difficult to factor tumor heterogeneity into treatment design.

Heterogeneity in the extracellular matrix, tumor dynamics, and immune compartments are current areas of active research, as these determinants of tumor growth and treatment resistance have not been given adequate consideration so far. For example, the role of collagen in brain tumor growth remains poorly understood, as are the factors that render these tumors resistant to immune checkpoint inhibitors. Equally, the dynamic nature of these tumors has consequences for our understanding of tumor invasion into healthy brain, and the interactions of immune cytotoxic lymphocytes with tumor cells. The invasive areas of the tumor border are also highly variable, demonstrating that heterogeneity needs to be considered across all tumor locations. Invasion is the major determinant of tumor progression and patient death, highlighting the importance of characterizing its histological, functional, molecular, vascular, and immune heterogeneity across the temporal spectrum. We predict that future therapeutic approaches will need to be effective across different tumor areas, spatially, functionally, and molecularly, to improve the overall treatment efficacy for GBM.

## Author Contributions

AC, SF, MV, TH, WA-H, YU, FN, SM, MC, and PL wrote the manuscript, with overall guidance, revisions, and edits from PL and MC. MV, SF, and AC prepared Figures. AC, SF, PL, and MC reviewed and edited the manuscript. All authors contributed to the article and approved the submitted version.

## Funding

This work was supported by the National Institutes of Health/ National Institute of Neurological Disorders & Stroke (NIH/ NINDS) Grants R21-NS091555, R37-NS094804, and R01-NS074387 to MC. R01-NS076991, R01-NS082311, and R01-NS096756 to PL. Rogel Cancer Center Scholar Award and Forbes Senior Research Scholar Award to MC. National institutes of Health/National Institute of Biomedical Imaging and Bioengineering (NIH/NIBIB) Grant R01-EB022563 to PL, and MC. University of Michigan MCube; the Department of Neurosurgery; the University of Michigan Rogel Comprehensive Cancer Center; the Pediatric Brain Tumor Foundation (BTF), Ian's Friends Foundation to PL and MC, Leah’s Happy Hearts Foundation, The Chad Tough Foundation, and the Biosciences Initiative in Brain Cancer to MC and PL. UL1 TR002240 to Michigan Institute for Clinical and Health Research (MICHR), Postdoctoral Translational Scholars Program (PTSP), Project F049768 to AC.

## Conflict of Interest

The authors declare that the research was conducted in the absence of any commercial or financial relationships that could be construed as a potential conflict of interest.

## Publisher’s Note

All claims expressed in this article are solely those of the authors and do not necessarily represent those of their affiliated organizations, or those of the publisher, the editors and the reviewers. Any product that may be evaluated in this article, or claim that may be made by its manufacturer, is not guaranteed or endorsed by the publisher.
